# Synthesis of Active Graphene with Para-Ester on Cotton Fabrics for Antistatic Properties

**DOI:** 10.3390/nano10061147

**Published:** 2020-06-11

**Authors:** Mengting Su, Xiaoting Chen, Liyuan Zhang, Jie Min

**Affiliations:** 1 College of Chemistry, Chemical Engineering and Biotechnology, Donghua University, 2999 North Renmin Road, Songjiang, Shanghai 201620, China; sissi.wood@gmail.com (M.S.); ximushuiyuan@126.com (X.C.); 2 National Local Joint Engineering Laboratory for Advanced Textile Processing and Clean Production, Science and Technology Institute, Wuhan Textile University, Wuhan 430200, China; liyuan.zhang@monash.edu; 3 Department of Chemical Engineering, Monash University, Wellington Road, Clayton 3800, Australia

**Keywords:** acid chloride, para-ester, active graphene, cotton, antistatic, conductive, fabrics

## Abstract

The excellent electrical properties of graphene provide a new functional finishing idea for fabricating conductive cotton fabrics with antistatic properties. This work develops a novel method for synthesizing active graphene to make cotton fabrics conductive and to have antistatic properties. The graphite was oxidized to graphene oxide (GO) by the Hummers method, and was further acid chlorinated and reacted with the para-ester to form the active graphene (JZGO). JZGO was then applied to cotton fabrics and was bonded to the fiber surface under alkaline conditions. Characterizations were done using FT-IR, XRD and Raman spectroscopy, which indicated that the para-ester group was successfully introduced onto JZGO, which also effectively improved the water dispersibility and reactivity of the JZGO. Furthermore, this study found that the antistatic properties of the fabric were increased by more than 50% when JZGO was 3% by weight under low-humidity conditions. The washing durability of the fabrics was also evaluated.

## 1. Introduction

Functional cotton fabrics are widely used in manufacture and daily life, and their antistatic property is one of their most important functions. This is because cotton fabrics at low temperatures and in humid environments risk causing sparks due to electrostatic discharge, which may lead to dangerous burning and explosion hazards [1]. Therefore cotton fabrics usually require an antistatic finishing process during manufacture, such as applying an antistatic agent (e.g., alkoxysilane, chitosan) to the surface [1,2]. It is also found that antistatic effects can be further enhanced by simply coating a layer of conductive compounds on the surface of cotton fabrics [3,4,5,6]. However, the simple coating or addition of antistatic agents cannot maintain the antistatic effect for long due to the problem of fading or being washed away, which is also called the fastness [7]. Therefore, the effort to develop a new method for fabricating conductive cotton fabrics combining good antistatic performance and fastness is attracting researchers worldwide.

To design such conductive cotton fabrics, a variety of conductive materials are studied. We found that graphene can be a good candidate, as it has good conductivity [8] and mechanical properties [4]. As a new type of green material, graphene has developed rapidly since it was discovered [9]. Researchers have prepared graphene using different methods, including stripping [10,11], redox [12,13,14], chemical vapor deposition (CVD) [15,16], and epitaxial growth [17,18]. Graphene has found various applications in the fields of sensing [19], supercapacitors [20], optics, green chemistry, wearable sensors [21,22,23], and for medical purposes [24]. It also found a potential to be used as an antistatic agent on cotton fabrics [25,26], as a small amount of graphene provides a high antistatic effect at low temperatures and in humid conditions [27,28].

Graphene does not have any functional groups on its surface, so it cannot be bonded effectively to fabrics to achieve a good fastness. It is also very easy to aggregate in solution [29], because of the strong interaction between the layers of the graphene. This causes poor compatibility of graphene with the matrix, and poor dispersibility in the solvent. To resolve these problems, different methods for modifying graphene have been proposed [30,31,32], including with non-covalent bonding [33,34,35] and covalent bonding [36,37,38].

In the dyeing industry, a successful example for using covalent bonding to improve the fastness of the dyes is to make the dyes become reactive to the cotton surface. These have been named “reactive dyes”. Reactive dyes consist of a chromophore, a bridging group, and other structures, e.g., a soluble group. To improve the bonding between graphene and cotton fibers and to achieve a good fastness of graphene on cotton, in this work, we ‘borrowed’ the idea of using a bridging group from reactive dyes to modify the graphene on cotton fiber surfaces with the para-ester vinyl sulfone ((2-((4-aminobenzene) sulfonyl) ethoxy) sulfonic acid)-based structure which has good water solubility and a high reactivity to cotton. Cotton fibers and reactive dyes are covalently bonded and have good fastness [39]. In particular, dyes with the para-ester vinyl sulfone-based structure have good water solubility and high reactivity [40]. Under alkaline conditions, the sulfate groups of the para-ester form the vinyl sulfone group by an elimination reaction [41]. This work aims to first modify graphene by reacting the para-ester with the graphene oxide to introduce the vinyl sulfone group; then to covalently bond the graphene to the nucleophilic group on cotton fibers with the vinyl sulfone groups to improve the antistatic properties and the fastness at the same time.

## 2. Materials and Methods

### 2.1. Materials

Natural flake graphite, potassium permanganate, hydrogen peroxide, sodium nitrate, 98% concentrated sulfuric acid, concentrated hydrochloric acid, dichlorosulfoxide, *N,N*-dimethylformamide, anhydrous sodium carbonate, anhydrous sodium sulfate, were all of analytical grade, purchased from Sinopharm Chemical Reagent Co., Ltd., Shanghai, China. p-β-hydroxyethyl sulfone aniline sulfate was obtained from Ji’nan Yumao Chemical Co., Ltd., Jinan, China. Cotton fabric (plain weave) was made by Zhejiang Shaoxing Pudun Textile Co., Ltd. Shaoxing, China. The standard soap sheet was purchased from Dongguan Dongcheng Baifei testing instrument factory, Dongguan, China.

### 2.2. Preparation of Graphene Oxide

**Figure 1:**
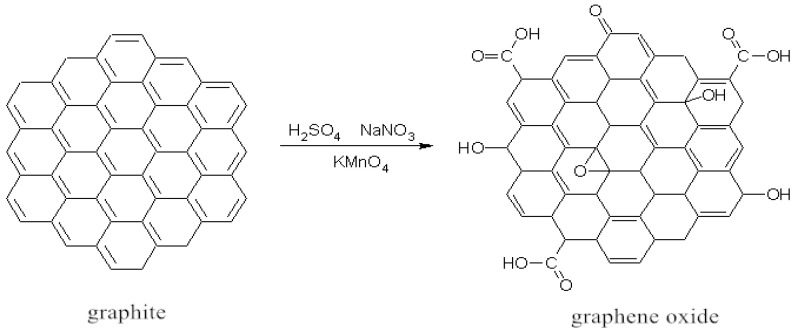
Reaction mechanism for synthesis of graphene oxide.

Two grams of natural flake graphite and 1 g of NaNO_3_ were added to 50 mL of concentrated H_2_SO_4_ (98%) in a three-necked flask with stirring and kept below 4 °C for 1 h. Then 6 g of KMnO_4_ (in batches, finished in 30 min) was added to the mixture, with the temperature below 10 °C and reacted for 2 h. The three-necked flask was transferred to a water bath with a constant temperature of 35 °C for 1 h. Ninety-two milliliters of deionized water was slowly added into the mixture and the temperature was increased and maintained at 95 °C for 30 min. Deionized water (200 mL) and H_2_O_2_ (10 mL) with the concentration of 30% were added to the mixture until no more bubbles were generated. The product was collected by filtration while it was still warm and washed with HCl of 10% and deionized water several times until the centrifuged supernatant was neutral. The precipitate of the product was removed and dispersed ultrasonically and dried for 24 h to obtain the product, graphene oxide (GO). Figure 1 illustrates the synthesis reaction mechanism.

### 2.3. Preparation of Active Graphene

**Figure 2:**
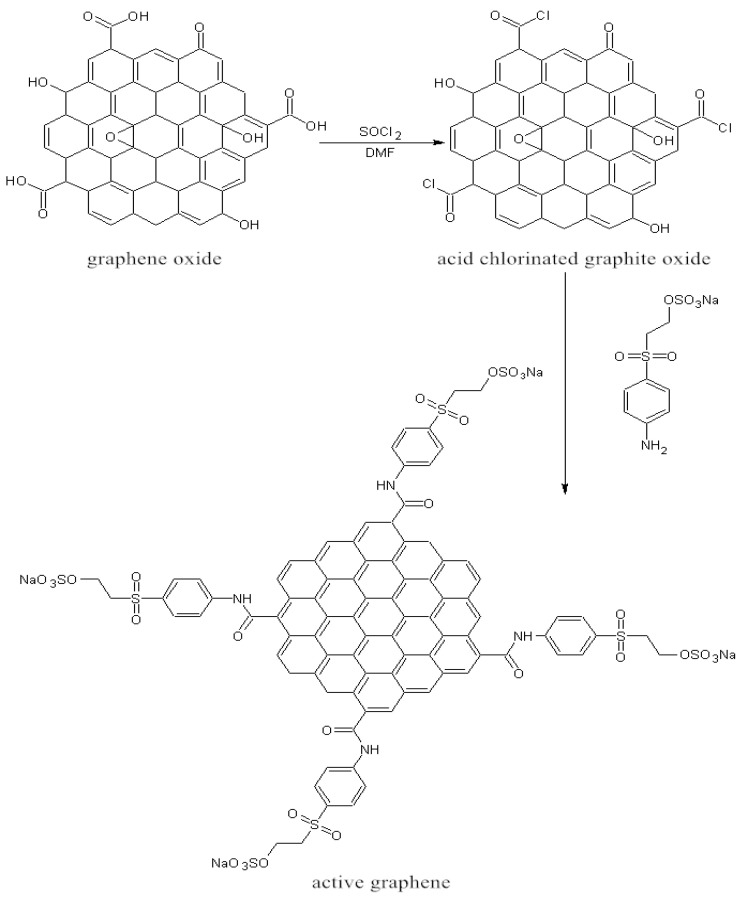
Reaction mechanism for synthesis of active graphene.

One hundred milligrams of graphene oxide was dispersed in 20 mL sodium sulfoxide (SOCl_2_), and then 0.5 mL of *N,N*-dimethylformamide (DMF) was added into the mixture. The mixture was heated up to 60 °C and kept for 24 h. After the reaction was completed, the temperature was increased to 90 °C, and the excess SOCl_2_ was removed to obtain acid chlorinated graphite oxide (GOCl). Half a gram of purified para-ester was then dissolved in 20 mL of DMF, before it was poured into the GOCl. The temperature of the reaction was kept at 90 °C to continue the reaction for another 24 h. After the reaction, the precipitates were washed with deionized water, collected, and then heated in an oven at 60 °C for 8 h to obtain active graphene (JZGO). The synthesis mechanism is illustrated in Figure 2.

### 2.4. Active Graphene Modification onto Cotton Fabric

**Figure 3:**
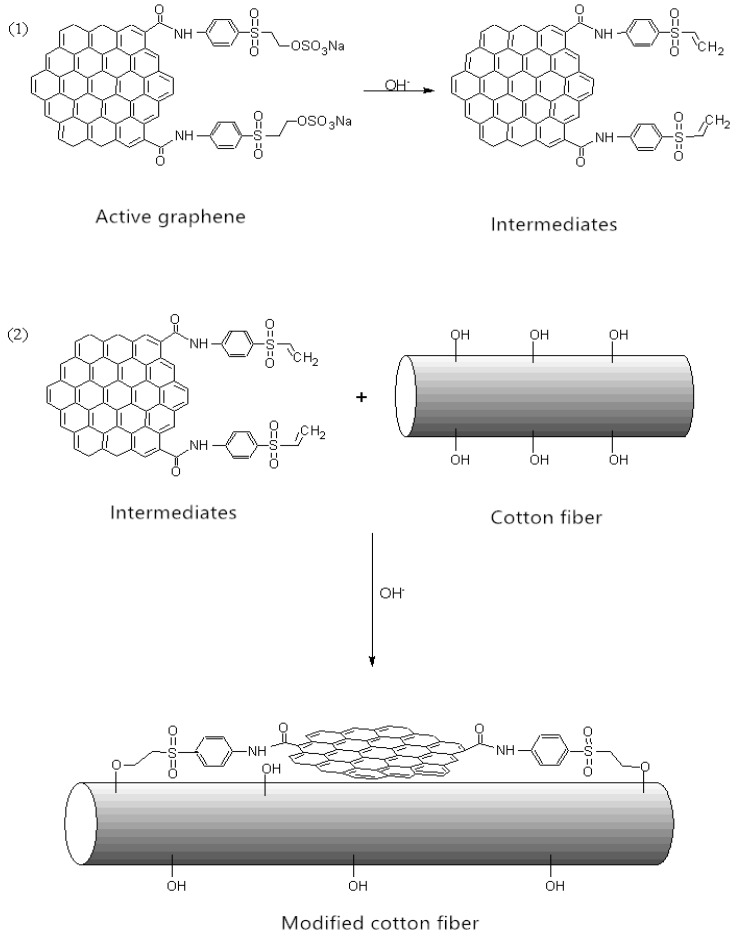
The reaction process of modified cotton fiber.

The reaction of active graphene with cotton fabric was divided into the following two parts: under the alkaline condition, the H atom on the α-carbon of the para-ester became active in the electron-withdrawing action of the sulfone group, and the elimination reaction was easily carried out with the sulfate group to form the vinyl sulfone group. Active graphene generated vinyl sulfone, which reacted with the hydroxyl through covalent bonding on cotton fiber in alkaline conditions, so that graphene tablets were grafted to cotton fabric. The reaction process is shown in Figure 3. The active graphene (JZGO) agent was then made of 1% and 3% (o.w.f = of the weight of fabrics) active graphene, and sodium carbonate (10 g/L). The bath ratio of reagent to water was 1:20. The traditional padding-dry-bake process [42] was used to apply the active graphene onto the surface of the cotton fabrics. The above active graphene JZGO was well dispersed in an ultrasonic bath for 20 min before it was applied on to the fabrics. Cotton fabrics were then dipped into the well-dispersed active graphene suspension, before they were put through rollers to remove the excess water. The fabrics with JZGO were dried in the oven at 60 °C, and further baked at 150 °C for 3 min. Post-treatments, including rinsing with cold water, soap, hot water, and cold water, were carried out before the final drying of the fabrics.

### 2.5. Characterization of Surface Modification

The surface functional groups of graphene oxide (GO) and active graphene (JZGO) were analyzed by a Varian 640 infrared spectrometer, Varian Co., Atlanta, GA, USA. The test wavelength range was 400–4000 cm^−1^, the test resolution was 4 cm^−1^, and the scanning frequency was 32 times. The samples were analyzed by inVia-Reflex laser microscopy Raman spectroscopy. The excitation wavelength was 532 nm and the test range was 1000–3500 cm^−1^.

The crystallite sizes of the samples (GO and JZGO) and the change of the interlayer distance between the samples before and after the reaction were measured by D/max-2550VB+/PC X-ray diffractometer, Rigalcu Co., Tokyo, Japan. The test uses Cu-Kα radiation, tube pressure 40 kV, tube flow 200 mA, wavelength *λ* = 1.54 Å, and scanning angle range of 5–90°. The surface morphology of the samples was characterized by a HITACHI / TM-1000 scanning electron microscope, HITACHI, Tokyo, Japan. The thermogravimetric curve of the sample was measured by a TG 209 F1 thermal analyzer, NETZSCH Co., Selb, Germany. The temperature range was from room temperature to 900 °C under a gas atmosphere of N_2_ with a gas flow rate of 10 mL/min.

### 2.6. Properties of Modified Cotton Fabrics

The antistatic properties of the fabric were measured by a YG (B) 342E fabric electrostatic tester, Wenzhou Darong Textile Instrument Co., Ltd., Wenzhou, China. The fabric areas were 45 mm × 45 mm, pre-dried at 50 °C for 30 min, and then placed in a condition of 40% humidity for 5 h. The sample was put into the instrument when its humidity was 40%. The static voltage data of each sample was measured three times. The fastness to soaping/washing was done using a soaping solution containing 5 g/L prepared with the standard soap sheet and 2 g/L of sodium carbonate, with the bath ratio of 1:50, washing for 30 min at a temperature of 60 °C.

## 3. Results and Discussions

### 3.1. Characterisation of the Synthesized Graphene Oxide (GO) and the Active Graphene (JZGO)

#### 3.1.1. Morphology of the Graphene Oxide and the Active Graphene

**Figure 4:**
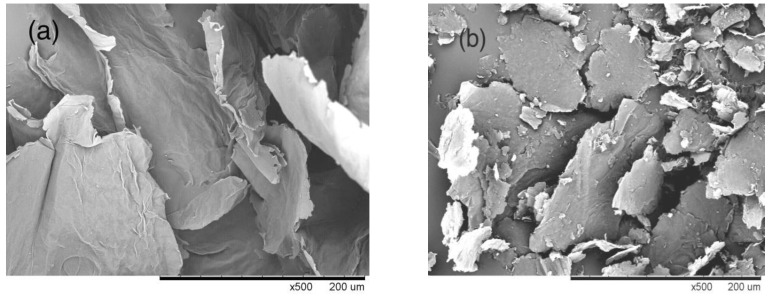
SEM images of (**a**) graphene oxide (GO); (**b**) active graphene (JZGO).

The SEM (Scanning Electron Microscope) image in Figure 4a shows the morphology of the graphene oxide (GO). The morphology of the GO shows a fluffy appearance with a large number of folds on the surface, as well as the edge curling and a large slice area. This was caused by the introduction of oxygen atoms. The oxidation reaction made the original flat graphite sheet surface wrinkled and the edge of the layer became curly. The surface morphology of the active graphene (JZGO) shown in Figure 4b is similar to that of GO, the surface of which is also wrinkled and has a granular para-ester.

#### 3.1.2. FT-IR, Raman and XRD Characterizations of the Graphene Oxide and the Active Graphene

**Figure 5:**
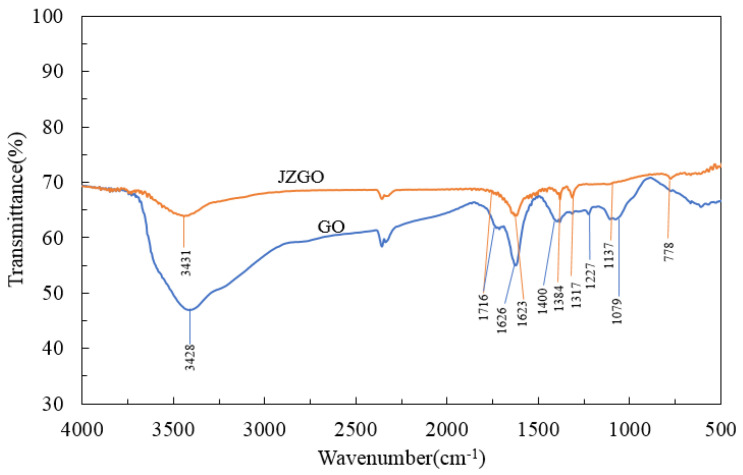
Infrared spectra of graphene oxide (GO) and active graphene (JZGO).

To further study the structure and the relationship between GO and JZGO, FT-IR, Raman and XRD characterizations were carried out. The infrared spectra of GO and JZGO were obtained and are compared in Figure 5. The spectra of the GO show main absorption peaks in the vicinity of 3428 cm^−1^, 1716 cm^−1^, 1626 cm^−1^, 1400 cm^−1^, 1227 cm^−1^ and 1079 cm^−1^. Here, 1628 cm^−1^ represents the stretching vibration of the carboxyl group C=O at the edge of the GO; 1626 cm^−1^ is the stretching vibration peak of C=C of the carbon ring; 1400 cm^−1^ is the bending vibration peak of hydroxyl –OH in GO; 1227 cm^−1^ is the stretching vibration peak of C–O–C on the GO surface; 1079 cm^−1^ is the stretching vibration peak of C–OH [33]. The spectra indicated that the oxygen-containing functional groups, such as carboxyl groups (–COOH), hydroxyl groups (–OH), and epoxy groups (C–O–C), were introduced to the graphite [33]. A large number of hydroxyl groups after oxidation were introduced to the surface and the edge of the graphite sheet. The carbon atoms (C = C) connected to hydroxyl groups turned into C–C. At the same time, the hydroxyl group was dehydrated into an epoxy group, and the hydroxyl group at the edge was converted to a carboxyl group. Adjacent carboxyl or carbonyl groups were decarboxylated, thereby removing a portion of the functional groups, and the carbon content of the GO was gradually reduced.

Active graphene (JZGO) showed absorption peaks near 3431 cm^−1^, 1716 cm^−1^, 1623 cm^−1^, 1384 cm^−1^, 1317 cm^−1^, 1137 cm^−1^, and 778 cm^−1^. Here, 3431 cm^−1^ is the stretching vibration peak of N–H; 1716 cm^−1^ is the stretching vibration peak of C=O; 1623 cm^−1^ is the stretching vibration peak of C=C; 1384 cm^−1^ is the stretching vibration peak of S=O; 1317 cm^−1^ is C–N stretching vibration peak; 1137 cm^−1^ is the asymmetric stretching vibration peak of –SO_2_–; and 778 cm^−1^ is the stretching vibration peak of S–O. This indicated that GO reacted with the para-ester.

**Figure 6:**
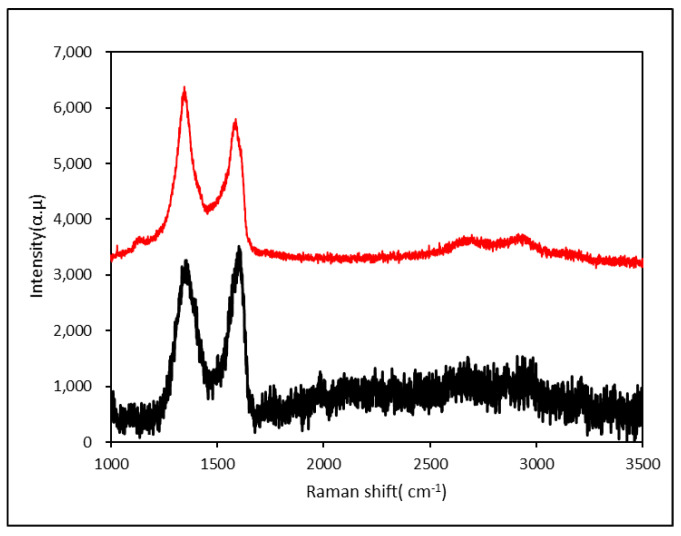
Raman spectra of the graphene oxide (GO) and activated graphene (JZGO).

As shown in Figure 6, the Raman spectra of graphene (GO) showed two characteristic peaks: 1354 cm^−1^ (D peak) and 1601 cm^−1^ (G peak). D peaks occurred when the graphite sample was defective or the Raman scattered light was collected at the disordered structure. After the oxidation of graphite, the G peak was broadened and the D peak was broadened and enhanced. This was because the carbon atom in the graphite sheet was bonded to the oxygen-containing group; and sp^3^ hybridization, a relatively disordered structure occurred, destroying the long-range order and symmetry of the graphite lattice. The degree of disorder is expressed as the ratio of the intensity of the D peak to the G peak, which is *R* = *I_D_*/*I_G_*. The intensity of the D peak of the JZGO increased and the peak width was narrowed down. The corresponding R value became larger, which means that the JZGO was more disordered than the GO. The more defects in the JZGO structure, the more subtropical carbon was added. This also indicated that the para-ester was grafted onto the GO [33].

**Figure 7:**
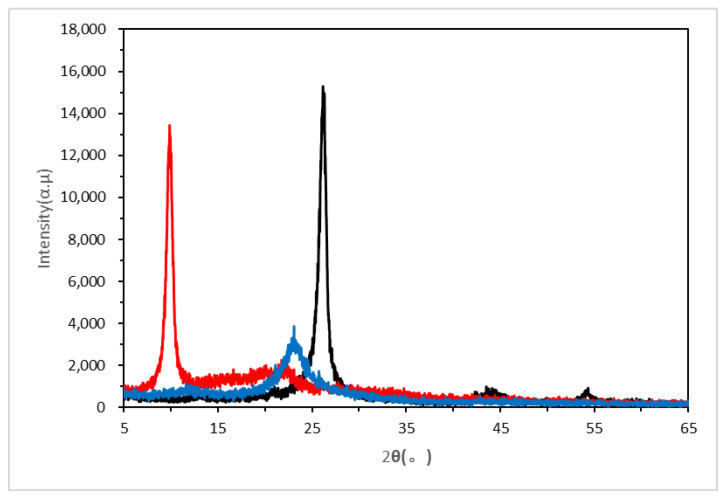
XRD spectrum of the graphite (G), graphene oxide (GO) and activated graphene (JZGO).

In Figure 7, the change of the characteristic diffraction peak reflects the transition from graphite to graphene oxide and from graphene oxide to active graphene. It can be seen from the curve of G (Graphene) that graphite exhibits a narrow and sharp characteristic diffraction peak at 2*θ* of 26.22°, representing the crystal interplanar spacing of *d* = 0.3396 nm, which suggests a typical graphite crystal structure. From the curve of GO (Graphene oxide), the characteristic diffraction peak of graphite disappears and a new diffraction peak appears at 2*θ* of 9.88°, which represents the crystal interplanar spacing of *d* = 0.8945 nm, indicating that due to the introduction of oxygen functional groups, the graphite hexagonal crystal structure was damaged, and the layer spacing of graphite lattice along the c-axis direction increased. These oxygen-containing groups were combined with water molecules through hydrogen bonds to impart hydrophilicity to the graphene, while further increasing the pitch of the graphite layer. JZGO showed a new diffraction peak with a weak intensity at 2*θ* of about 23°, and the spacing of the crystal layer was much lower than that of GO, but it was higher than natural flake graphite.

#### 3.1.3. Dispersion and Thermal Stability Characterizations

**Figure 8:**
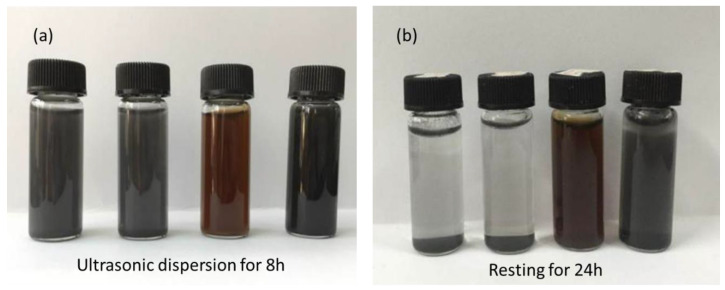
Dispersion of graphite, oxidized expanded graphite, graphene oxide, and active graphene dispersion, from left to right respectively, in: (**a**) Ultrasonic dispersion for 8 h; (**b**) resting for 24 h.

As shown in Figure 8a, after 8 h of ultrasound treatment the dispersion became homogenous, while after resting for 24 h (Figure 8b), most of natural flake graphite and oxidized expanded graphite had settled. In contrast, the GO and JZGO were still evenly dispersed without precipitation. This was because the surface of the GO contained a large number of oxygen-containing functional groups such as carboxyl groups (–COOH), hydroxyl groups (–OH) and epoxy groups (C–O–C), which made the GO more hydrophilic and easy to disperse. For the same reason, the carboxyl group in the active graphene (JZGO) was converted into a sulfonic acid group (–SO_3_H) and made the JZGO also easily dispersed.

**Figure 9:**
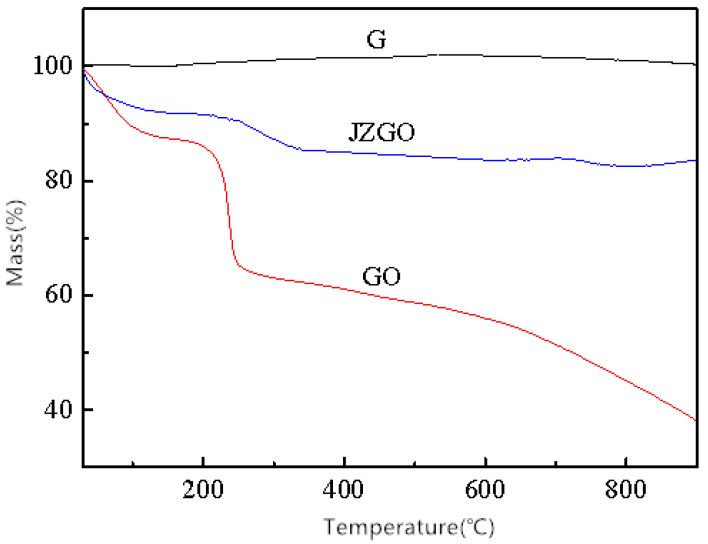
TG curves of graphite (G), graphene oxide (GO) and activated graphene (JZGO).

In Figure 9, the oxidation occurred at three stages when significant mass loss was observed, where 20 to 200 °C corresponds to the desorption process of free water and combined water in GO. At 220 to 300 °C, the weight loss of GO was very rapid due to the decomposition of oxygen-containing functional groups, like hydroxyl (–OH), epoxy (C–O–C) which became small molecules of gas, e.g., carbon dioxide and water vapor. After this, weight loss became slower because the remainder was mainly the relatively stable GO carbon skeleton. Above 700 °C, the pyrolysis of carbon structure started, which damaged the carbon frame structure of the graphite through strong oxidation, thereby decreasing its thermal stability.

The JZGO lost mass at two stages: the first stage (20 to 250 °C) was the desorption process of free water and bound water, and the second stage (250 to 360 °C) was mainly caused by the oxygen-containing functional group and the grafted ester of the JZGO. From this part of the mass loss rate, the introduction amount of the para-ester on the JZGO was roughly estimated to be about 4%. Compared to the graphene oxide (GO), the weight loss rate of the JZGO was much smaller. This was because the amount of the oxygen-containing functional groups of JZGO, like hydroxyl (–OH), epoxy (C–O–C), was much less than in GO. Therefore, the thermal stability of JZGO was significantly improved. The reason why JZGO had a lesser amount of oxygen-containing functional groups was due to the acyl chloride reaction for preparing the JZGO. During the reaction, the strong dehydrating agent, thionyl chloride (SOCl_2_) had reacted with some carboxyl groups (–COOH), hydroxyl (–OH) and other groups which resulted in the reduction of the amount of the oxygen-containing functional groups and thus the thermal stability of the JZGO was improved.

### 3.2. Characterisation of the Modified Cotton Fabrics with Active Graphene

#### 3.2.1. Morphology of the Modified Cotton Fabrics

**Figure 10:**
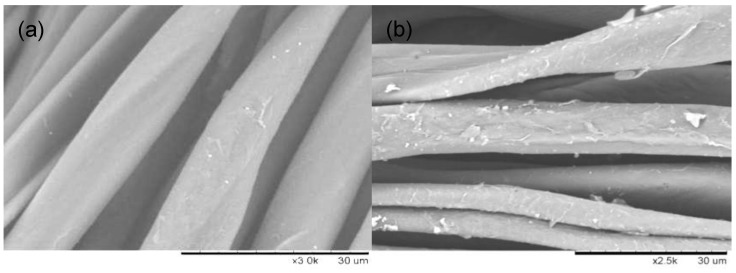
Scanning electron microscopy of cotton fabrics: (**a**) Before modification; (**b**) after modification.

The morphology of the modified cotton fabrics with the active graphene is shown in the SEM image of Figure 10. The cotton fabric surface was smooth before the modification reaction (Figure 10a), while the morphology of the modified cotton fabric surface was rough (Figure 10b), because the sheet of graphite was covered by the cotton fiber surface.

#### 3.2.2. Characterizations of the Antistatic Properties of the Modified Cotton Fabrics

**Figure 11:**
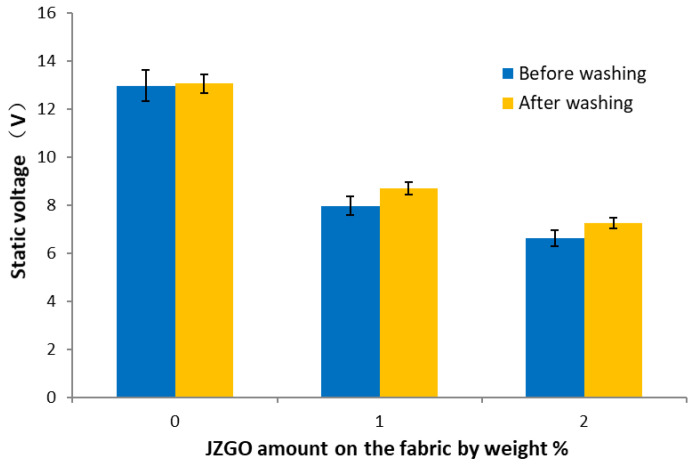
Static voltage of cotton fabric: JZGO-modified cotton fabric before washing (blue bars); JZGO-modified cotton fabric after 3 washings with soap (yellow bars).

As shown in Figure 11, the active graphene-modified cotton fabric showed a significant improvement on the antistatic effects in low humidity conditions. When adding 1% of active graphene, the static voltage of cotton fabric decreased from 13.0 V to 8.0 V; if the amount of active graphene increased to 3%, the static voltage of cotton fabric was reduced by about 50%, and the antistatic effect of cotton fabric was greatly improved. The static voltage was maintained at 7.3 V after three soap washings, which might be owing to the covalent bond of active graphene and cotton fabric.

## 4. Conclusions

In this study, the GO was prepared by the traditional Hummers method, and the carboxyl group was activated by acyl chloride reaction, then further reacted with the para-ester to obtain JZGO. Compared with GO, the JZGO was further disordered, and the interlayer spacing was further reduced, which was due to the introduction of a sulfonic acid group with good hydrophilic properties. In the process of acid chlorination, the oxygen-containing functional groups in graphene oxide was also removed, which gave JZGO better thermal stability. The JZGO reacted with the hydroxyl through covalent bonding on cotton fiber in alkaline conditions. In this research, JZGO was coated onto the cotton fabric using the conventional padding-baking process. When the amount of JZGO was up to 3% (o.w.f), the antistatic effect of cotton fabric was significantly improved. The active graphene-modified cotton fabric achieved good antistatic properties under low humidity conditions, and good fastness to washing.
